# Epstein–Barr virus encephalitis with excessive daytime sleepiness as the main manifestation: Two case reports

**DOI:** 10.1097/MD.0000000000030327

**Published:** 2022-08-26

**Authors:** Hui Zhao, Xiaobo Zhang, Haifeng Yang, Jun Gu

**Affiliations:** a Department of Neurology, Rudong County People’s Hospital, Nantong, China; b Department of Neurology, The First People’s Hospital of Changde City, Changde, China.

**Keywords:** case report, Epstein–Barr virus encephalitis, excessive daytime sleepiness, sleep awakening

## Abstract

Excessive daytime sleepiness (EDS) is a clinical manifestation of various disorders. Here, we report 2 cases of EDS related to Epstein–Barr virus (EBV) encephalitis.

**Patient concerns:**

Both the patients were elderly men. Case 1 presented with EDS with headache and fever. Case 2 was presented with EDS only. The 2 patients slept normally at night without taking sleeping pill. They were able to get up and go to the toilet and eat by themselves during the day, but they almost slept at other times.

**Diagnosis:**

After admission, a lumbar puncture was performed to collect the cerebrospinal fluid, and next-generation sequencing showed that EBV infection was detected. Combined with the patient’s head magnetic resonance imaging and clinical features, a diagnosis of EBV encephalitis was made.

**Interventions:**

Both patients received antiviral therapy.

**Outcomes:**

Case 1 had a rapid improvement in headache and fever and was discharged from the hospital after the symptoms of EDS gradually improved. In case 2, EDS symptoms gradually improved. Two patients were followed up for 3 months after discharge, and the outcome was good.

**Lessons:**

EDS can also be the main clinical manifestation of viral encephalitis, and we should diagnose and identify it early and treat it promptly.

## 1. Introduction

Excessive daytime sleepiness (EDS) manifests as higher daytime sleep pressure and a tendency to increase sleep duration. The etiology of EDS varies and includes sleep deprivation, sleep-disordered breathing, circadian rhythm sleep-wake disorders, and central disorders of hypersomnolence (narcolepsy, idiopathic hypersomnia, and Klein–Levine syndrome). Additionally, EDS may represent the symptoms of an underlying medical or psychiatric disorder.^[1]^ The clinical symptoms of EBV encephalitis vary, ranging from mild to severe, with no obvious specificity. The current case reports are mostly on children, and there are relatively few adult cases. This article introduces 2 cases of EBV encephalitis with EDS as the main manifestation.

## 2. Case introduction

Written informed consent was obtained from both patients.

### 2.1. Case 1

**Figure 1.**
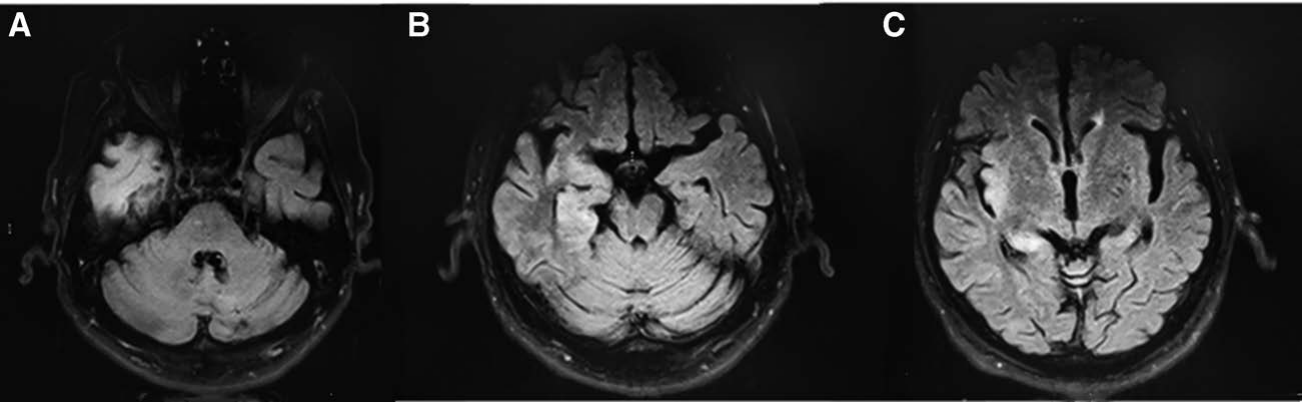
Brain magnetic resonance T2 FLAIR sequence of patient 1 (A) anterior hyperintensity in the right temporal lobe. (B) hyperintensity in the right amygdala and hippocampus. (C) hyperintensity in the right insula and hippocampus. T2 FLAIR = T2 fluid-attenuated inversion recovery.

A 69-year-old man with a history of hypertension, diabetes, and coronary heart disease. He came to the hospital in February 2021 because of excessive sleepiness for 3 days. The patient was almost asleep except for eating and going to the toilet and had headache, dizziness, fever, and no abnormal mental behavior. Physical examination revealed a body temperature of 38.1°C, drowsiness, ability to answer correctly, soft neck, normal muscle strength of limbs, and negative pathological signs. After admission, high signal intensity in the right temporal lobe, insula, amygdala, and hippocampus was observed on the fluid-attenuated inversion recovery sequence of the cranial magnetic resonance imaging (MRI; Fig. 1). The intracranial pressure was measured by lumbar puncture at 70 mm H_2_O, cerebrospinal fluid examination showed a nucleated cell count of 9 × 10^6^/L, and Pan test was weakly positive. Cerebrospinal fluid biochemical cerebrospinal fluid protein level was 1050.7 mg/L. All 12 cases of cerebrospinal fluid autoimmune encephalitis were negative. Next-generation sequencing of cerebrospinal fluid suggested a human herpesvirus type 4 (EBV) sequence number of 13. Tumor marker levels were normal. The patient was diagnosed with EBV encephalitis. Antiviral therapy was administered via an intravenous infusion of ganciclovir. The patient’s headache and fever symptoms rapidly improved, and the drowsiness symptoms gradually improved. Half a month later, the patient underwent a lumbar puncture again, which showed that the cerebrospinal fluid white blood cell count and protein level were both lower than before. The patient’s follow-up outcomes after discharge were good.

### 2.2. Case 2

**Figure 2.**
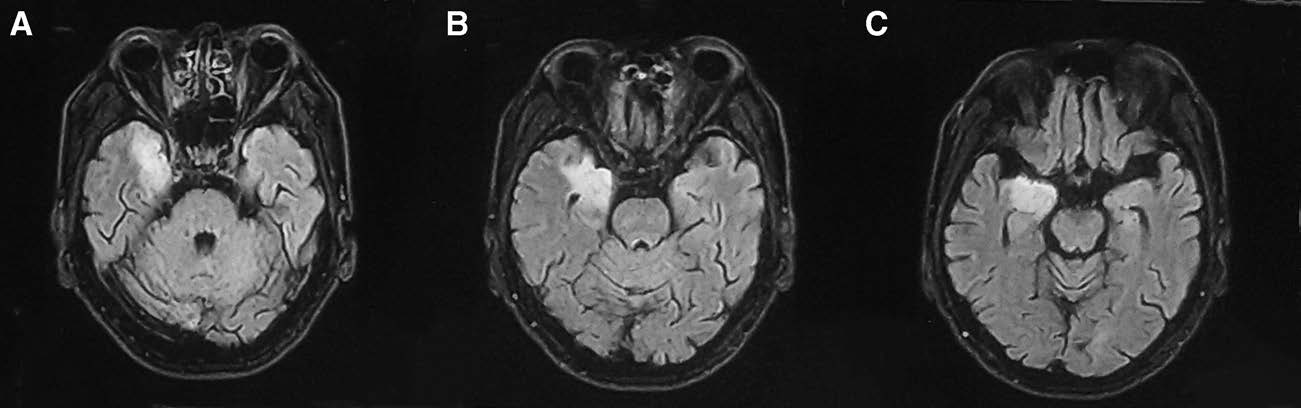
Brain magnetic resonance T2 FLAIR sequence of patient 2 (A) high signal in the right medial temporal lobe. (B, C) High signal in the right medial temporal lobe and amygdala. T2 FLAIR = T2 fluid-attenuated inversion recovery.

A 65-year-old man with a history of hypertension and diabetes. Due to excessive sleepiness for 20 days, he came to the hospital for treatment on November 2021. The patient slept for >20 hours a day, was still sleepy during the day, spoke less, was unconscious, had no abnormal behavior, and had no fever. There was no sleep paralysis, hypnagogic hallucinations, or cataplexy. MRI of the head revealed lesions in the right temporal lobe and amygdala (Fig. 2). Metabolic disease and limbic encephalitis should also be considered. A 24-hour video electroencephalogram showed no typical abnormal discharges or focal delta waves. Physical examination revealed a body temperature of 36.5°C, drowsiness, ability to answer correctly, soft neck, normal muscle strength of the limbs, and negative pathological signs. After admission, lumbar puncture was performed to measure intracranial pressure of 130 mm H_2_O, routine leukocytes of cerebrospinal fluid: 89 × 10^6^/L, mononuclear cells:88/89, Pan test: 1+, and cerebrospinal fluid biochemical cerebrospinal fluid protein: 1700 mg/L. Next-generation sequencing of cerebrospinal fluid suggested human herpesvirus 4 (EBV), with a sequence number of 4, and an identification confidence of 99%. Cerebrospinal fluid autoimmune encephalitis antibody tests were negative. Blood thyroid function and tumor indicators were normal. The patient was diagnosed with EBV encephalitis. Intravenous infusion of ganciclovir was administered. The patient’s drowsiness symptoms improved, the white blood cell count and protein level in the cerebrospinal fluid decreased, and the patient had a good follow-up after discharge.

## 3. Discussion

Both patients in this study were elderly men with subacute onset, and the main clinical manifestation was EDS. Brain MRI showed different degrees of involvement of the limbic system in both cases. Cerebrospinal fluid showed increased white blood cells and protein, and the pathogenic microorganisms were confirmed to be infected by the EBV by metagenomic detection of pathogenic microorganisms. Based on the patient’s clinical symptoms, head magnetic resonance imaging, and cerebrospinal fluid examination results, considering that the diagnosis of EBV encephalitis was clear, the symptoms improved after antiviral treatment and he was discharged from the hospital, and his condition was stable during follow-up.

The EBV belongs to the gamma subtype of the herpes virus and is a DNA virus that exists widely in nature. EBV encephalitis is an uncommon complication of EBV infection and is usually limited to children and immunocompromised patients. EBV encephalitis and other viral encephalitis lack specificity in clinical symptoms, but encephalitis caused by herpes virus is more common in adults, and the acute onset mostly manifests as fever, headache, mental disorders, and disturbance of consciousness. Epileptic seizures can have subacute or chronic onset. The main symptom of the 2 patients in this study was EDS, which is relatively rare.

MRI of both patients showed involvement of the medial temporal lobe and limbic lobe. The limbic system includes structures, such as the hippocampus, amygdala, insula, and cingulate gyrus. It holds the functions of participates in learning and memory, regulating visceral activities, emotional cognition, and sleep-wake. Common clinical symptoms of limbic encephalitis are memory loss, abnormal mental behavior, and epilepsy. There are numerous causes of limbic encephalitis, which are generally divided into infectious and noninfectious disease. Infectious herpes simplex encephalitis is the most common, while other types of viral encephalitis are relatively rare.^[2]^ Adults infected with EBV encephalitis are more common in immunocompromised patients, and EBV encephalitis after allogeneic hematopoietic stem cell transplantation has been reported.^[3]^ Noninfectious diseases are mostly immune- or tumor-related. In this study, second-generation sequencing of cerebrospinal fluid confirmed that both patients had EBV infection. Case 1 was accompanied by a headache and fever. Both patients tested negative for autoimmune brain antibodies in the cerebrospinal fluid. After antiviral treatment, the symptoms improved, and there is insufficient evidence of immune-related limbic encephalitis.

The main circadian rhythm control point in humans is the suprachiasmatic nucleus in the front of the hypothalamus, which promotes the production of hypothalamic secretin (orexin) through a circuit, and the hypothalamic secretin affects the serotonin nerve of the dorsal raphe nucleus,^[4]^ noradrenergic neurons in the nucleus locus coeruleus,^[5]^ histaminergic neurons in the papillary nucleus of the tubercle,^[6]^ cholinergic neurons in the basal forebrain^[7]^ that play an excitatory role. In addition, cholinergic neurons in the pedunculopontine nucleus, laterodorsal tegmental nucleus, and glutamatergic neurons in the parabrachial nucleus promote wakefulness.^[8]^ The structures that promote sleep mainly include the ventrolateral preoptic nucleus^[9]^ and the middle preoptic nucleus^[10]^ of the hypothalamus, which are excited during sleep and the pineal gland that produces melatonin.^[11]^ Therefore, the main neurons that regulate sleep arousal are located in the brainstem and hypothalamus. However, the common sites of herpesvirus encephalitis are the medial temporal lobe, frontal orbital surface, and limbic system. The 2 patients in this study failed to find a responsible lesion associated with EDS on MRI. The affected medial temporal lobes and hippocampus in our 2 patients were associated with memory consolidation during sleep.^[12,13]^

Unfortunately, owing to limited conditions, we did not perform multiple sleep latency tests for the 2 patients and did not measure the level of orexin in the cerebrospinal fluid to further determine the cause of EDS in the patients. The regulatory mechanism of sleep arousal and circadian rhythm is very complex and has not yet been fully explored. For example, type 1 narcolepsy is characterized by decreased hypocretin levels. In addition, studies have found that individuals carrying HLA-DQB1*06:02 have a much greater risk of developing the disease than do noncarriers.^[14]^ The presence of streptococcal infection may initiate or catalyze an autoimmune response against hypocretin cells in narcolepsy.^[15]^ H1N1 influenza and related Pandemrix vaccinations have been suggested as possible specific environmental triggers for narcolepsy episodes.^[16,17]^ It can be seen that genetic susceptibility, immune response, and environmental factors are all related to the pathogenesis of the disease. There are several causes of drowsiness that require further study.

## 4. Summary

In the face of encephalitis in clinical practice, it is difficult to determine the type of pathogenic microorganisms using routine and biochemical cerebrospinal fluid. We are often limited to empirical treatment or combinations of antibiotics. Imaging studies can also lead to misdiagnosis, for example, some herpes simplex encephalitis may result in unilateral brain MRI lesions, similar to acute ischemic stroke, and may sometimes be considered intracranial space-occupying lesions. With EDS as the main manifestation of this type of atypical encephalitis, next-generation cerebrospinal fluid sequencing technology has improved the ability to accurately type the virus, allowing us to further understand the clinical characteristics of various subtypes of herpesvirus encephalitis. The report in this paper provides a reference for the treatment of the disease in clinical practice, which is helpful for clinicians in understanding the pathogenesis of the disease, increasing the understanding of the disease, reducing misdiagnosis or missed diagnosis, and making early correct treatment.

## Author contributions

Conceptualization: Hui Zhao, Xiaobo Zhang, Jun Gu.

Investigation: Hui Zhao, Haifeng Yang.

Methodology: Hui Zhao, Xiaobo Zhang, Jun Gu.

Writing—original draft: Hui Zhao.

Writing—review & editing: Hui Zhao, Haifeng Yang, Jun Gu.
